# 3-Meth­oxy-4-[3-(2-methyl-4-nitro-1*H*-imidazol-1-yl)prop­oxy]benzaldehyde

**DOI:** 10.1107/S1600536809031894

**Published:** 2009-08-15

**Authors:** Lei Jin, Guang-Zhou Wang, Cheng-He Zhou

**Affiliations:** a Laboratory of Bioorganic & Medicinal Chemistry, School of Chemistry and Chemical Engineering, Southwest University, Chongqing 400715, People’s Republic of China; b School of Pharmaceutical Sciences, Southwest University, Chongqing 400715, People’s Republic of China

## Abstract

In the title mol­ecule, C_15_H_17_N_3_O_5_, the dihedral angle between the benzene and imidazole rings is 3.69 (2)°. The crystal structure is stabilized by weak inter­molecular C—H⋯O hydrogen bonds and π–π stacking inter­actions with a centroid–centroid distance of 3.614 (1) Å.

## Related literature

For general background to the biological activity of nitro­imidazole and its derivatives, see:Demirayak *et al.* (1999 ▶); Huang *et al.* (2007 ▶); Olender *et al.* (2009 ▶). For the synthetic procedure, see: Khalafi-Nezhad *et al.* (2005 ▶).
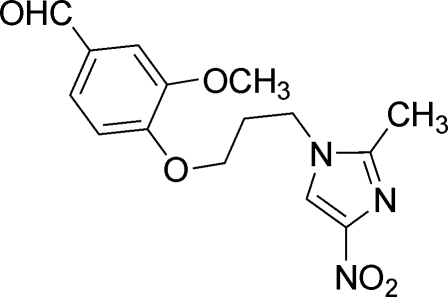


## Experimental

### 

#### Crystal data

C_15_H_17_N_3_O_5_
*M* *_r_* = 319.32
Monoclinic, 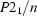

*a* = 9.4885 (14) Å
*b* = 13.048 (2) Å
*c* = 12.745 (2) Å
β = 101.120 (3)°
*V* = 1548.3 (4) Å^3^
*Z* = 4
Mo *K*α radiation
μ = 0.10 mm^−1^
*T* = 173 K
0.28 × 0.24 × 0.2 mm

#### Data collection

Bruker SMART CCD diffractometer
Absorption correction: multi-scan (*SADABS*; Sheldrick, 1996 ▶) *T*_min_ = 0.971, *T*_max_ = 0.979
7761 measured reflections
3329 independent reflections
2329 reflections with *I* > 2σ(*I*)
*R*_int_ = 0.022

#### Refinement

*R*[*F* ^2^ > 2σ(*F* ^2^)] = 0.043
*wR*(*F* ^2^) = 0.130
*S* = 1.03
3329 reflections
210 parameters
H-atom parameters constrained
Δρ_max_ = 0.27 e Å^−3^
Δρ_min_ = −0.19 e Å^−3^

### 

Data collection: *SMART* (Bruker, 2000 ▶); cell refinement: *SAINT* (Bruker, 2000 ▶); data reduction: *SAINT*; program(s) used to solve structure: *SHELXS97* (Sheldrick, 2008 ▶); program(s) used to refine structure: *SHELXL97* (Sheldrick, 2008 ▶); molecular graphics: *DIAMOND* (Brandenburg & Putz, 1999 ▶); software used to prepare material for publication: *SHELXTL* (Sheldrick, 2008 ▶).

## Supplementary Material

Crystal structure: contains datablocks I, global. DOI: 10.1107/S1600536809031894/lh2876sup1.cif

Structure factors: contains datablocks I. DOI: 10.1107/S1600536809031894/lh2876Isup2.hkl

Additional supplementary materials:  crystallographic information; 3D view; checkCIF report

## Figures and Tables

**Table 1:** Hydrogen-bond geometry (Å, °)

*D*—H⋯*A*	*D*—H	H⋯*A*	*D*⋯*A*	*D*—H⋯*A*
C4—H4*B*⋯O4^i^	0.98	2.58	3.415 (3)	144
C8—H8⋯O4^ii^	0.95	2.51	3.229 (2)	133
C10—H10*B*⋯O2^iii^	0.99	2.56	3.312 (2)	133
C10—H10*A*⋯O1^iv^	0.99	2.58	3.461 (2)	148
C14—H14⋯O1^iv^	0.95	2.29	3.166 (2)	153

Symmetry codes: (i) 
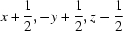
; (ii) 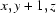; (iii) 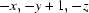; (iv) 
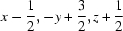
.

**Fig. 1.**
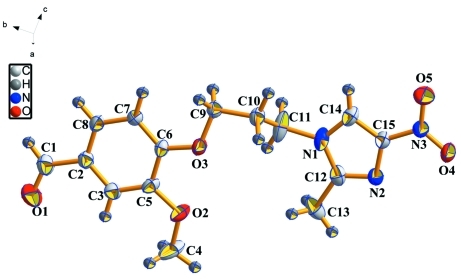
The molecular structure of (I), showing the atom-numbering scheme. Displacement ellipsoids are drawn at the 50% probability level.

## supplementary crystallographic information

### Comment

Nitroimidazole and its derivatives possess several biological activities such as radiosensitizer, anti-tuberculosis and antimicrobial (Demirayak *et al., *1999; Huang *et al.*, 2007; Olender *et al.*, 2009). In view of the therapeutic potentials of nitroimidazole derivatives, we are interested in the research and development of nitroimidazole compounds as drugs. Herein we report the crystal structure of the title compound (I).

The stucture of the title compound (I) is shown in Fig 1. In the molecule the dihedral angle between the benzene and imidazole rings is 3.69 (2)°. The crystal structure is stabilized by weak intermolecular C—H···O hydrogen bonds and significant π–π stacking interactions with a centroid to centroid ditance of 3.614 (1)Å between benzene and imidazole rings related by the symmetry operator (1/2-x, 1/2+y, 1/2-z).

### Experimental

Compound (I) was synthesized according to the procedure of Khalafi-Nezhad *et al.* (2005). Single crystals used in X-ray diffraction studies were grown by slow evaporation at room temperature of solutions of (I) in ethyl acetate and dichlormethane mixtures.

### Refinement

Hydrogen atoms were placed in calculated positions with C—H = 0.95Å (aromatic), 0.99Å (methylene) and 0.98Å (methyl) with *U*iso(H) = 1.2*U*eq(C) (aromatic and methylene C) or 1.5*U*eq(C) (methyl C).

### Crystal data

**Table:**

C_15_H_17_N_3_O_5_	*F*(000) = 672
*M**_r_* = 319.32	*D*_x_ = 1.370 Mg m^−^^3^
Monoclinic, *P*2_1_/*n*	Mo *K*α radiation, λ = 0.71073 Å
Hall symbol: -P 2yn	Cell parameters from 3149 reflections
*a* = 9.4885 (14) Å	θ = 2.3–26.9°
*b* = 13.048 (2) Å	µ = 0.10 mm^−^^1^
*c* = 12.745 (2) Å	*T* = 173 K
β = 101.120 (3)°	Block, colorless
*V* = 1548.3 (4) Å^3^	0.28 × 0.24 × 0.2 mm
*Z* = 4	

### Data collection

**Table:**

Bruker SMART CCD diffractometer	3329 independent reflections
Radiation source: fine-focus sealed tube	2329 reflections with *I* > 2σ(*I*)
graphite	*R*_int_ = 0.022
φ and ω scans	θ_max_ = 27.0°, θ_min_ = 2.3°
Absorption correction: multi-scan (*SADABS*; Sheldrick, 1996)	*h* = −7→12
*T*_min_ = 0.971, *T*_max_ = 0.979	*k* = −15→16
7761 measured reflections	*l* = −13→16

### Refinement

**Table:**

Refinement on *F*^2^	Primary atom site location: structure-invariant direct methods
Least-squares matrix: full	Secondary atom site location: difference Fourier map
*R*[*F*^2^ > 2σ(*F*^2^)] = 0.043	Hydrogen site location: inferred from neighbouring sites
*wR*(*F*^2^) = 0.130	H-atom parameters constrained
*S* = 1.03	*w* = 1/[σ^2^(*F*_o_^2^) + (0.0623*P*)^2^ + 0.3992*P*] where *P* = (*F*_o_^2^ + 2*F*_c_^2^)/3
3329 reflections	(Δ/σ)_max_ < 0.001
210 parameters	Δρ_max_ = 0.27 e Å^−^^3^
0 restraints	Δρ_min_ = −0.19 e Å^−^^3^

### Special details

**Table:**

Geometry. All e.s.d.'s (except the e.s.d. in the dihedral angle between two l.s. planes) are estimated using the full covariance matrix. The cell e.s.d.'s are taken into account individually in the estimation of e.s.d.'s in distances, angles and torsion angles; correlations between e.s.d.'s in cell parameters are only used when they are defined by crystal symmetry. An approximate (isotropic) treatment of cell e.s.d.'s is used for estimating e.s.d.'s involving l.s. planes.
Refinement. Refinement of *F*^2^ against ALL reflections. The weighted *R*-factor *wR* and goodness of fit *S* are based on *F*^2^, conventional *R*-factors *R* are based on *F*, with *F* set to zero for negative *F*^2^. The threshold expression of *F*^2^ > σ(*F*^2^) is used only for calculating *R*-factors(gt) *etc*. and is not relevant to the choice of reflections for refinement. *R*-factors based on *F*^2^ are statistically about twice as large as those based on *F*, and *R*- factors based on ALL data will be even larger.

### Fractional atomic coordinates and isotropic or equivalent isotropic displacement parameters (Å^2^)

**Table:**

	*x*	*y*	*z*	*U*_iso_*/*U*_eq_	
C1	0.2645 (2)	1.03088 (14)	−0.03024 (16)	0.0502 (5)	
H1	0.2344	1.0901	0.0028	0.060*	
C2	0.21471 (17)	0.93177 (12)	0.00141 (13)	0.0363 (4)	
C3	0.25719 (18)	0.84045 (12)	−0.04102 (14)	0.0381 (4)	
H3	0.3193	0.8421	−0.0912	0.046*	
C4	0.3482 (2)	0.65169 (17)	−0.1102 (2)	0.0691 (7)	
H4A	0.3107	0.6882	−0.1769	0.104*	
H4B	0.3681	0.5803	−0.1262	0.104*	
H4C	0.4370	0.6847	−0.0737	0.104*	
C5	0.20870 (17)	0.74857 (12)	−0.00975 (13)	0.0346 (4)	
C6	0.11391 (16)	0.74686 (11)	0.06315 (12)	0.0305 (3)	
C7	0.07388 (17)	0.83703 (11)	0.10519 (13)	0.0322 (4)	
H7	0.0114	0.8359	0.1551	0.039*	
C8	0.12529 (17)	0.92983 (12)	0.07433 (13)	0.0347 (4)	
H8	0.0985	0.9921	0.1038	0.042*	
C9	−0.01627 (18)	0.64126 (12)	0.16566 (14)	0.0360 (4)	
H9A	−0.1090	0.6775	0.1440	0.043*	
H9B	0.0344	0.6699	0.2346	0.043*	
C10	−0.04042 (17)	0.52728 (12)	0.17563 (14)	0.0378 (4)	
H10A	−0.1107	0.5152	0.2225	0.045*	
H10B	−0.0800	0.4981	0.1043	0.045*	
C11	0.0990 (2)	0.47573 (13)	0.2218 (2)	0.0616 (6)	
H11A	0.1721	0.4966	0.1802	0.074*	
H11B	0.1316	0.4999	0.2962	0.074*	
C12	0.16148 (17)	0.29675 (13)	0.16772 (13)	0.0384 (4)	
C13	0.2589 (2)	0.33135 (16)	0.09670 (16)	0.0560 (5)	
H13A	0.2936	0.2717	0.0624	0.084*	
H13B	0.3407	0.3680	0.1390	0.084*	
H13C	0.2066	0.3772	0.0417	0.084*	
C14	0.01304 (17)	0.30668 (12)	0.28026 (15)	0.0403 (4)	
H14	−0.0475	0.3304	0.3264	0.048*	
C15	0.04200 (16)	0.20834 (11)	0.25747 (13)	0.0326 (4)	
N1	0.09014 (14)	0.36345 (10)	0.22222 (12)	0.0417 (4)	
N2	0.13429 (14)	0.20076 (10)	0.18821 (11)	0.0346 (3)	
N3	−0.01634 (15)	0.11998 (11)	0.29957 (12)	0.0404 (4)	
O1	0.34061 (19)	1.04390 (12)	−0.09476 (13)	0.0758 (5)	
O2	0.24432 (13)	0.65445 (9)	−0.04271 (11)	0.0493 (4)	
O3	0.06945 (12)	0.65190 (8)	0.08547 (10)	0.0376 (3)	
O4	0.02197 (15)	0.03485 (9)	0.27613 (12)	0.0544 (4)	
O5	−0.10418 (15)	0.13411 (11)	0.35800 (12)	0.0611 (4)	

### Atomic displacement parameters (Å^2^)

**Table:**

	*U*^11^	*U*^22^	*U*^33^	*U*^12^	*U*^13^	*U*^23^
C1	0.0631 (12)	0.0379 (10)	0.0515 (11)	−0.0115 (8)	0.0156 (10)	0.0021 (8)
C2	0.0422 (9)	0.0306 (8)	0.0360 (9)	−0.0055 (7)	0.0074 (7)	0.0004 (7)
C3	0.0385 (9)	0.0378 (9)	0.0404 (9)	−0.0086 (7)	0.0139 (7)	−0.0058 (7)
C4	0.0556 (12)	0.0577 (13)	0.1071 (19)	−0.0074 (10)	0.0486 (13)	−0.0360 (13)
C5	0.0327 (8)	0.0294 (8)	0.0427 (9)	−0.0027 (6)	0.0097 (7)	−0.0106 (7)
C6	0.0306 (8)	0.0248 (7)	0.0355 (8)	−0.0027 (6)	0.0046 (6)	−0.0010 (6)
C7	0.0370 (8)	0.0285 (8)	0.0327 (8)	0.0011 (6)	0.0106 (7)	−0.0008 (6)
C8	0.0420 (9)	0.0249 (8)	0.0365 (9)	0.0012 (6)	0.0057 (7)	−0.0022 (6)
C9	0.0391 (9)	0.0279 (8)	0.0435 (9)	−0.0007 (7)	0.0140 (7)	0.0017 (7)
C10	0.0375 (9)	0.0284 (8)	0.0483 (10)	−0.0015 (7)	0.0106 (7)	0.0036 (7)
C11	0.0487 (11)	0.0249 (9)	0.0984 (17)	−0.0025 (8)	−0.0175 (11)	0.0050 (9)
C12	0.0367 (8)	0.0364 (9)	0.0391 (9)	−0.0067 (7)	−0.0004 (7)	0.0046 (7)
C13	0.0567 (12)	0.0595 (12)	0.0517 (12)	−0.0214 (10)	0.0102 (10)	0.0122 (10)
C14	0.0356 (8)	0.0316 (9)	0.0529 (11)	0.0045 (7)	0.0065 (8)	−0.0035 (8)
C15	0.0321 (8)	0.0273 (8)	0.0384 (9)	0.0018 (6)	0.0068 (7)	0.0007 (6)
N1	0.0360 (8)	0.0254 (7)	0.0597 (10)	−0.0008 (6)	−0.0008 (7)	0.0048 (6)
N2	0.0368 (7)	0.0309 (7)	0.0370 (7)	−0.0023 (6)	0.0092 (6)	0.0003 (6)
N3	0.0445 (8)	0.0319 (7)	0.0494 (9)	0.0022 (6)	0.0202 (7)	0.0034 (6)
O1	0.1041 (12)	0.0577 (9)	0.0781 (11)	−0.0285 (9)	0.0487 (10)	0.0027 (8)
O2	0.0480 (7)	0.0329 (7)	0.0749 (9)	−0.0048 (5)	0.0318 (7)	−0.0182 (6)
O3	0.0431 (6)	0.0236 (6)	0.0494 (7)	−0.0037 (5)	0.0176 (5)	−0.0026 (5)
O4	0.0732 (9)	0.0259 (6)	0.0739 (9)	0.0032 (6)	0.0383 (7)	0.0049 (6)
O5	0.0638 (9)	0.0543 (8)	0.0784 (10)	0.0023 (7)	0.0471 (8)	0.0018 (7)

### Geometric parameters (Å, °)

**Table:**

C1—O1	1.207 (2)	C9—H9B	0.9900
C1—C2	1.460 (2)	C10—C11	1.499 (2)
C1—H1	0.9500	C10—H10A	0.9900
C2—C8	1.374 (2)	C10—H10B	0.9900
C2—C3	1.400 (2)	C11—N1	1.468 (2)
C3—C5	1.371 (2)	C11—H11A	0.9900
C3—H3	0.9500	C11—H11B	0.9900
C4—O2	1.428 (2)	C12—N2	1.315 (2)
C4—H4A	0.9800	C12—N1	1.371 (2)
C4—H4B	0.9800	C12—C13	1.483 (2)
C4—H4C	0.9800	C13—H13A	0.9800
C5—O2	1.3615 (18)	C13—H13B	0.9800
C5—C6	1.412 (2)	C13—H13C	0.9800
C6—O3	1.3567 (18)	C14—C15	1.355 (2)
C6—C7	1.376 (2)	C14—N1	1.356 (2)
C7—C8	1.390 (2)	C14—H14	0.9500
C7—H7	0.9500	C15—N2	1.362 (2)
C8—H8	0.9500	C15—N3	1.428 (2)
C9—O3	1.4304 (19)	N3—O4	1.2235 (17)
C9—C10	1.514 (2)	N3—O5	1.2339 (18)
C9—H9A	0.9900		
			
O1—C1—C2	125.53 (19)	C9—C10—H10A	109.7
O1—C1—H1	117.2	C11—C10—H10B	109.7
C2—C1—H1	117.2	C9—C10—H10B	109.7
C8—C2—C3	120.47 (14)	H10A—C10—H10B	108.2
C8—C2—C1	118.55 (15)	N1—C11—C10	113.74 (14)
C3—C2—C1	120.98 (16)	N1—C11—H11A	108.8
C5—C3—C2	119.63 (15)	C10—C11—H11A	108.8
C5—C3—H3	120.2	N1—C11—H11B	108.8
C2—C3—H3	120.2	C10—C11—H11B	108.8
O2—C4—H4A	109.5	H11A—C11—H11B	107.7
O2—C4—H4B	109.5	N2—C12—N1	111.65 (15)
H4A—C4—H4B	109.5	N2—C12—C13	125.48 (17)
O2—C4—H4C	109.5	N1—C12—C13	122.86 (16)
H4A—C4—H4C	109.5	C12—C13—H13A	109.5
H4B—C4—H4C	109.5	C12—C13—H13B	109.5
O2—C5—C3	125.59 (15)	H13A—C13—H13B	109.5
O2—C5—C6	114.59 (14)	C12—C13—H13C	109.5
C3—C5—C6	119.82 (14)	H13A—C13—H13C	109.5
O3—C6—C7	125.37 (14)	H13B—C13—H13C	109.5
O3—C6—C5	114.55 (13)	C15—C14—N1	104.31 (15)
C7—C6—C5	120.08 (14)	C15—C14—H14	127.8
C6—C7—C8	119.74 (14)	N1—C14—H14	127.8
C6—C7—H7	120.1	C14—C15—N2	112.96 (14)
C8—C7—H7	120.1	C14—C15—N3	125.07 (15)
C2—C8—C7	120.23 (14)	N2—C15—N3	121.97 (13)
C2—C8—H8	119.9	C14—N1—C12	107.49 (13)
C7—C8—H8	119.9	C14—N1—C11	125.74 (17)
O3—C9—C10	105.75 (12)	C12—N1—C11	126.73 (16)
O3—C9—H9A	110.6	C12—N2—C15	103.59 (13)
C10—C9—H9A	110.6	O4—N3—O5	123.37 (14)
O3—C9—H9B	110.6	O4—N3—C15	119.10 (13)
C10—C9—H9B	110.6	O5—N3—C15	117.52 (13)
H9A—C9—H9B	108.7	C5—O2—C4	116.73 (14)
C11—C10—C9	109.72 (14)	C6—O3—C9	118.76 (12)
C11—C10—H10A	109.7		

### Hydrogen-bond geometry (Å, °)

**Table:**

*D*—H···*A*	*D*—H	H···*A*	*D*···*A*	*D*—H···*A*
C4—H4B···O4^i^	0.98	2.58	3.415 (3)	144
C8—H8···O4^ii^	0.95	2.51	3.229 (2)	133
C10—H10B···O2^iii^	0.99	2.56	3.312 (2)	133
C10—H10A···O1^iv^	0.99	2.58	3.461 (2)	148
C14—H14···O1^iv^	0.95	2.29	3.166 (2)	153

Symmetry codes: (i) *x*+1/2, −*y*+1/2, *z*−1/2; (ii) *x*, *y*+1, *z*; (iii) −*x*, −*y*+1, −*z*; (iv) *x*−1/2, −*y*+3/2, *z*+1/2.
